# Cut-off points for anthropometric indices to screen for hypertension among Iranian adults of the Bandare-Kong cohort: a cross-sectional study

**DOI:** 10.1186/s12889-022-14489-8

**Published:** 2022-11-11

**Authors:** Abnoos Azarbad, Tayebe Aghnia, Abdullah Gharibzadeh, Shideh Rafati, Seyyed Mohammad Hashemi, Hasan Zarei, Masoumeh Kheirandish

**Affiliations:** 1 Endocrinology and Metabolism Research Center, Hormozgan University of Medical Sciences, Bandar Abbas, Iran; 2 Student Research Committee, Faculty of Medicine, Hormozgan University of Medical Sciences, Bandar Abbas, Iran; 3 Cardiovascular Research Center, Hormozgan University of Medical Sciences, Bandar Abbas, Iran; 4 Social Determinants in Health Promotion Research Center, Hormozgan University of Medical Sciences, Bandar Abbas, Iran

**Keywords:** Hypertension, Blood pressure, Anthropometric indices, Prospective Epidemiological Research Studies in IrAN (PERSIAN)

## Abstract

**Background:**

Obesity is one of the major determinants of blood pressure. This study aimed to determine the optimal sex- and age-specific cut-off points of anthropometric indices, including body mass index (BMI), waist circumference (WC), hip circumference (HC), wrist circumference (WrC), waist-hip ratio (WHR), and waist-height ratio (WHtR), to screen for hypertension (HTN) in a cohort of Iranian adults aged 35 to 70 years, and to compare the predictive performance of the indices based on receiver operating characteristic (ROC) curves.

**Methods:**

This population-based study was carried out on the participants aged 35 to 70 years of the Bandare-Kong Non-Communicable Diseases (BKNCD) Cohort Study, a part of the Prospective Epidemiological Research Studies in IrAN (PERSIAN). Using the area under the receiver operating characteristic curve (AUC) and Youden's J index, optimal sex- and age-specific cut-off points of the anthropometric indices in association with HTN were calculated.

**Results:**

This study included a total of 2256 females and 1722 males. HTN was diagnosed in 736 females (32.6%) and 544 males (31.6%). The optimal cut-off of WC for HTN was 90 cm in males and 95 cm in females, with an area under the ROC curve (AUROC) of 0.60 and 0.64, respectively. For HC, the optimal cut-off was 95 cm for males and 108 cm for females (AUROC = 0.54 for both). Moreover, WrC optimal cut-offs were 17 cm for males (AUROC = 0.56) and 15 cm for females (AUROC = 0.57). As for BMI, the optimal cut-off was 25 kg/m^2^ in males and 27 kg/m^2^ in females (AUROC of 0.59 and 0.60, respectively). Also, a cut-off of 0.92 was optimal for WHR in males (AUROC = 0.64) and 0.96 in females (AUROC = 0.67). On the other hand, WHtR optimal cut-offs were 0.52 for males and 0.60 for females (AUROC of 0.63 and 0.65, respectively).

**Conclusions:**

WHR and WHtR, as anthropometric indices of obesity, were demonstrated to be significant predictors of HTN. Further, we suggest using WHR (cut-off point of 0.92 for males and 0.96 for females) and WHtR (cut-off point of 0.52 for males and 0.60 for females) as measures of preference to predict HTN among the southern Iranian population. Further multicenter longitudinal studies are recommended for a more accurate prediction of HTN.

## Background

Non-communicable diseases (NCDs) impose an enormous yet growing burden globally. The prevalence of NCDs has risen worldwide, affecting both males and females and all age groups [1]. Globally, NCDs account for 41 million annual deaths or approximately 71% of overall mortality; however, according to WHO, in 2019, this figure rose to 74% [2, 3]. NCDs claim the lives of over 15 million people from the ages of 30 to 69 every year; 85% of these deaths take place in countries with low and middle incomes. NCDs are responsible for 57% of deaths in the Eastern Mediterranean Region [4]. Hypertension (HTN) is one of NCDs' most critical risk factors [5]. It is highly prevalent globally, and by 2025, HTN is estimated to affect more than 1.56 billion people worldwide, up from 972 million in the year 2000 [6]. In 2019, HTN contributed to 10.8 million deaths worldwide [7]. Approximately two-thirds of patients with HTN live in developing countries [8, 9]. According to a recent meta-analysis conducted in Iran, a developing Eastern Mediterranean country, the prevalence of HTN was found to be 25% among females and 24% among males [10].

Obesity is one of the major determinants of blood pressure. There have been various explanations as to how body fat distribution affects blood pressure and HTN risk [11–13]. Anthropometric indices are essential for detecting obesity at an early stage. The anthropometric indices of obesity can be divided into two categories: measures of general obesity, including the body mass index (BMI), and measures of central obesity, such as waist circumference (WC), hip circumference (HC), and their ratios, waist-hip ratio (WHR) and waist-height ratio (WHtR). It is uncertain whether anthropometric indices of general or central obesity have a stronger correlation with blood pressure [14–17].

According to previous research [18], BMI used to be the primary determinant of HTN, as it was found to be more reliable than WC and WHtR. Some studies have found WC to be a more accurate screening tool than BMI, WHR, and WHtR [19–23]. Other studies have found WHR to be more reliable at predicting HTN than BMI, WC, and WHtR [24]. Furthermore, there have been several recent studies where WHtR has been found to have better predictive abilities than WHR, BMI, and WC among females and males [25–31].

Another noteworthy finding was that, in a recent systematic review conducted to assess the effectiveness of anthropometric indices in predicting cardiovascular diseases (CVDs), abdominal obesity indices, particularly WHR, led to a better prediction of CVD prevalence in adults [32]. Further, a study conducted among western Iranian adults proposed that BMI is a better predictor of metabolic syndrome than WC and WHR [33].

Given the existing controversy regarding the best anthropometric index for predicting HTN, this study aimed to determine the optimal sex- and age-specific cut-off points of anthropometric indices (BMI, WC, HC, WrC, WHR, and WHtR) to screen for HTN in a cohort of Iranian adults aged 35 to 70 years, and to compare the predictive performance of the indices based on receiver operating characteristic (ROC) curves.

## Methods

### Study design and sampling

The dataset for this descriptive population-based cross-sectional study was obtained from the Bandare-Kong Non-Communicable Diseases (BKNCD) research (*N* = 4063), a large-scale prospective study conducted between November 2016 and November 2018, among people aged 35–70, as part of the Prospective Epidemiological Research Studies in Iran (PERSIAN). The precise study design and methodologies have already been discussed in depth [34]. Among the study subjects, pregnant women and incomplete data were excluded. In total, 3978 participants were analyzed in this study.

### Data collection

The participants were interviewed by trained interviewers using a comprehensive standardized questionnaire for collecting demographics, medical and drug history.

### Anthropometric measurements

A transportable weighing scale, with a precision of 0.01 kg was used to weigh the participants while minimally clothed and without shoes. The subjects' heights were measured using a height rod, with 0.5 cm precision, while standing barefoot and with shoulders relaxed and arms at sides. BMI was obtained by dividing the subjects' weight in kilograms by their height in meters square. WC was measured using a retractable tape in the horizontal plane halfway between the lowest rib and the top of the iliac crest, with an accuracy of 0.1 cm. HC was measured parallel to the floor, with the arms relaxed at the sides and the maximum circumference over the buttocks. Wrist circumference (WrC) was measured with subjects in a seated position from both wrists using a tape meter positioned over the Lister tubercle of the distal radius and over the distal ulna, and an average was taken. WHR and WHtR were obtained by dividing waist to hip and height, respectively.

### Blood pressure measurement

A calibrated mercury sphygmomanometer was used to measure systolic and diastolic blood pressure (SBP, DBP). Prior to measurement, the participants rested for 5 min while seated. Blood pressure was measured twice at a 15-min interval from the right arm. The mean of the two consecutive blood pressure measurements was then utilized to compute the SBP and DBP that were used in the analysis. HTN was defined as SBP ≥ 140 mmHg and/or DBP ≥ 90 mmHg, self-reported HTN, as well as individuals those received HTN treatments [34].

#### Statistical analysis

It was assumed that the distribution was normal because of the large sample size. While continuous variables were described using means and standard deviations (SD), categorical variables were described using numbers and percentages (%). When comparing variables stratified by sex (male/female) or age (age < 50 / age ≥ 50), t-tests and chi-square tests were used to compare continuous variables and categorical variables, respectively.

Receiver operating characteristic (ROC) curves were used to determine the diagnostic accuracy of the anthropometric indices and their optimal cut-off values for each sex and age group (age < 50/age ≥ 50). The area under the ROC curve (AUC) was calculated to assess the diagnostic ability of the anthropometric indices to predict HTN. An anthropometric measure with AUC = 1 discriminated clearly between hypertensive and non-hypertensive individuals. However, an AUC of 0.5 indicated no difference in anthropometric values between the two groups, meaning the predictive power was invalid. Using Youden's J index, appropriate cut-off values were determined (maximum [sensitivity + specificity—1]).

Using the set cut-off values from the study, the anthropometric measures were dichotomized and assessed for their relationship with HTN using binary logistic regression following adjustment for age, physical activity, socio-economic status, smoking, and hookah consumption. Plus, all regression analyses were stratified according to sex. The ROC analysis was carried out using MedCalc software, and the other analyses were conducted by SPSS version 25. A *P*-value of 0.05 or lower was regarded as statistically significant.

## Results

This study included 1722 (43.3%) males and 2256 (56.7%) females. Their age ranged from 35 to 70 years, with a mean (SD) of 48.3 (9.4) years. 544 males (31.6%) and 736 females (32.6%) were diagnosed with HTN. Table 1 displays the subjects' characteristics.

**Table 1:** Characteristics of participants according to sex

Characteristics	Total (*N* = 3978)	Male(*N* = 1722)	Female (*N* = 2256)	*P*-value
Age (year)	48.3(9.40)	48.3 (9.58)	48.2 (9.26)	0.722^a^
< 50	2322(58.4%)	1010(25.4%)	1312(33%)	0.770^b^
≥ 50	1656(41.6%)	712(17.9%)	944(23.7%)	
Residence				
Urban	3350(85.2%)	1468(37.3%)	1882(47.9%)	0.077^b^
Rural	582(14.8%)	232(5.9%)	350(8.9%)	
Marital status				
Single	93(2.4%)	21(0.5%)	72(1.8%)	< 0.001^b^
Married	3519(89.5%)	1662(42.3%)	1857(47.2%)	
Widow/divorced	320(8.1%)	17(0.4%)	303(7.7%)	
Education (years)				
≤ 5 years	2360(60.0%)	758(19.3%)	1602(40.7%)	< 0.001^b^
6–12 years	1247(31.7%)	740(18.8%)	507(12.9%)	
≥ 13 years	325(8.3%)	202(5.1%)	123(3.1%)	
Socio-economic status				
Low	1505(38.4%)	529(13.5%)	976(24.9%)	< 0.001^b^
Moderate	1656(42.3%)	766(19.6%)	890(22.7%)	
High	754(19.3%)	397(10.1%)	357(9.1%)	
Physical activity score (METs/ day)				
Low (24-36.5)	1021(26.0%)	462(11.8%)	559(14.2%)	< 0.001^b^
Moderate (36.6-44.9)	2338(59.5%)	902(23.0%)	1436(36.5%)	
Vigorous (>= 45)	570(14.5%)	334(8.5%)	236(6.0%)	
Hookah consumption				
Yes	693(17.6%)	389(9.9%)	304(7.7%)	< 0.001^b^
No	3239(82.4%)	1311(33.3%)	1928(49.0%)	
Cigarette smoking				
Yes	357(9.1%)	351(8.9%)	6(0.2%)	< 0.001^b^
No	3575(90.9%)	1349(34.3%)	2226(56.6%)	
Waist Circumference (cm)	93.8(11.9)	90.4 (11.2)	96.3 (11.8)	< 0.001^a^
Hip Circumference(cm)	100.1 (9.7)	97.8 (8.3)	101.9 (10.3)	< 0.001^a^
Wrist Circumference(cm)	16.5 (1.5)	17.2 (1.3)	16.0 (1.4)	< 0.001^a^
Body Mass Index (kg/m^2^)	26.9(5.0)	25.8(4.5)	27.78(5.2)	< 0.001^a^
Waist to Hip Ratio	0.9(0.6)	0.9(0.1)	0.9(0.1)	< 0.001^a^
Waist to Height Ratio	0.5(0.1)	0.5(0.1)	0.6(0.1)	< 0.001^a^
Systolic Blood Pressure (mmHg)	118.7(17.4)	120.5(16.4)	117.3(17.9)	< 0.001^a^
Diastolic Blood Pressure (mmHg)	76.9(10.3)	78.4(9.9)	75.9(10.5)	< 0.001^a^

Categorical variables are expressed by number and percentage (%) and continuous variables by the mean and standard deviation (SD)

^a^Independent-samples T-test

^b^Chi-square test

**Fig. 1:**
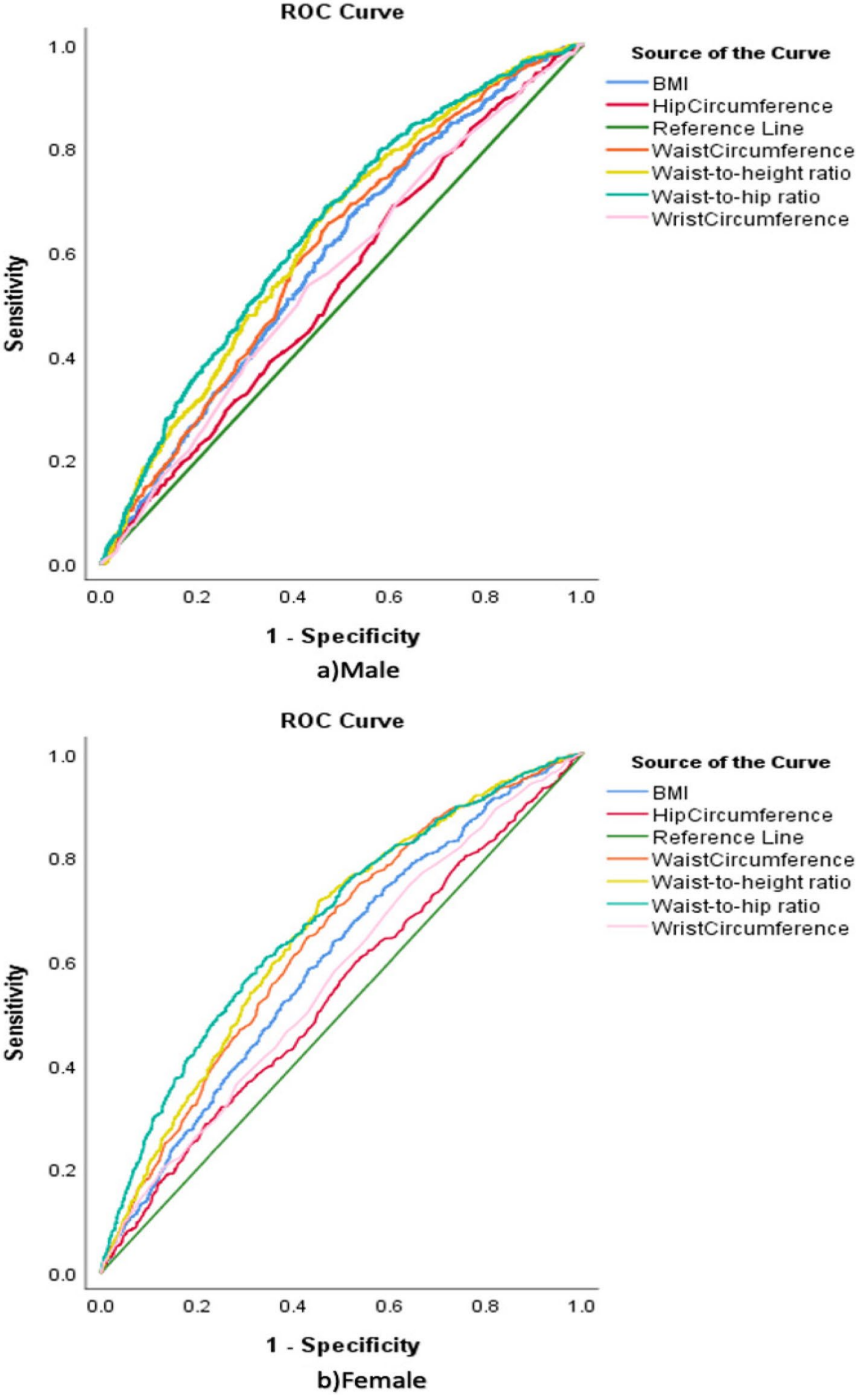
ROC curve displaying the ability of anthropometric indices to predict hypertension according to sex

**Fig. 2:**
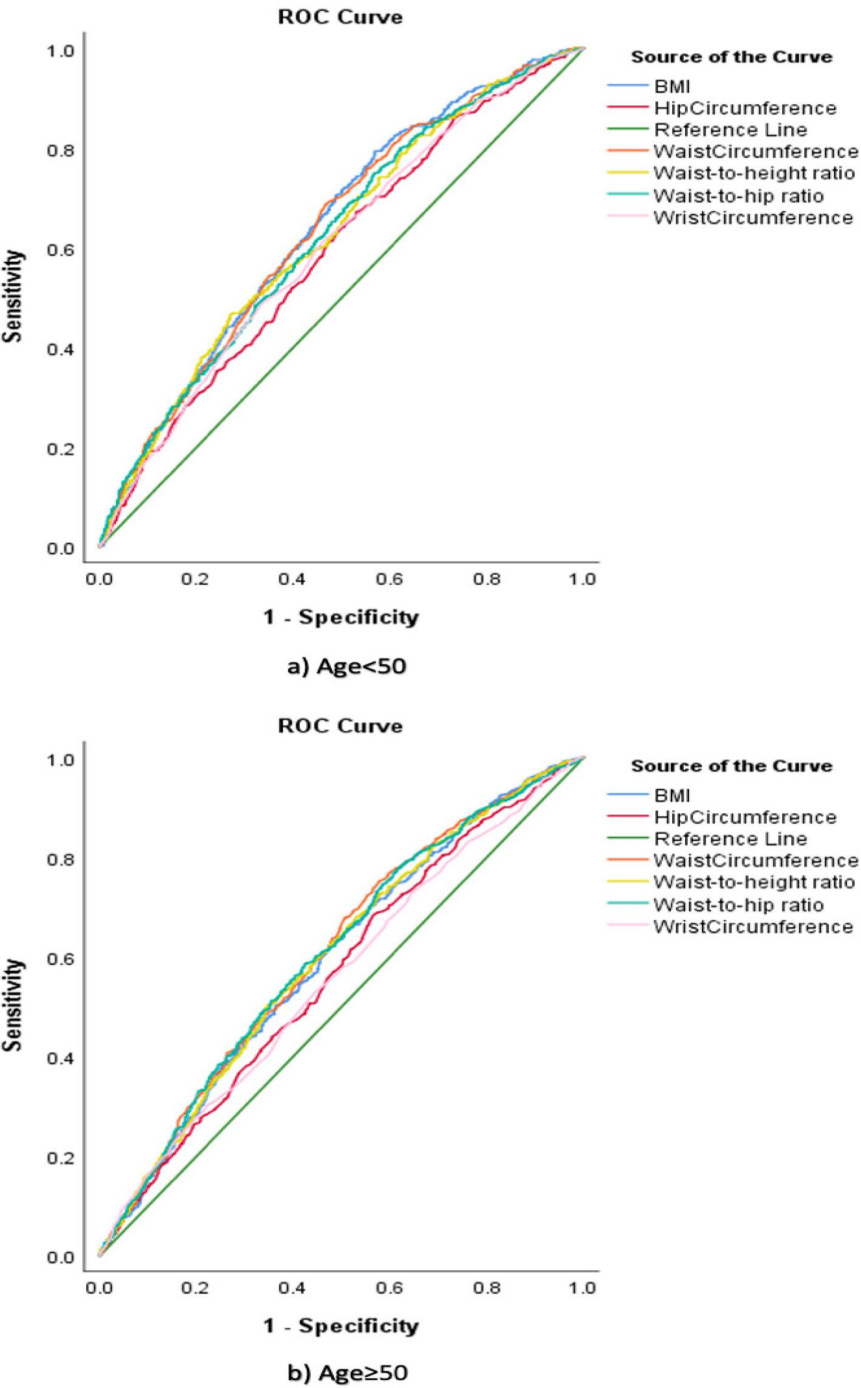
ROC curve displaying the ability of anthropometric indices to predict hypertension according to age

ROC analysis (Fig. 1) revealed that WHR and WHtR had better performance than other indices in females and males. According to Fig. 2, all anthropometric indices performed fairly similarly in predicting diagnosed HTN for age < 50 and age ≥ 50.

Table 2 displays the area under the ROC curve for males and females. WHR and WHtR showed better accuracy than other anthropometric indices in predicting HTN in females (AUC = 0.67 for WHR, AUC = 0.65 for WHtR) and males (AUC = 0.64 for WHR, AUC = 0.63 for WHtR).

**Table 2:** Area under the curve and optimal cut-off points demonstrating the ability of anthropometric indices to predict hypertension according to sex

Measures	Male					Female				
	**AUC (99% CI)**	*P* value*	**Cut-off value**	**Sensitivity**	**Specificity**	**AUC (99% CI)**	*P* value*	**Cut-off value**	**Sensitivity**	**Specificity**
Waist Circumference (cm)	0.60 (0.57–0.64)	< 0.001	90.0	66%	53%	0.64 (0.61–0.67)	< 0.001	96.0	65%	57%
Hip Circumference (cm)	0.54 (0.50–0.58)	< 0.001	95.0	69%	39%	0.54 (0.51–0.57)	< 0.001	108.0	29%	78%
Wrist Circumference (cm)	0.56 (0.52–0.60)	< 0.001	17.0	53%	57%	0.57 (0.54–0.60)	< 0.001	15.0	74%	36%
Body Mass Index (kg/m^2^)	0.59 (0.55–0.63)	< 0.001	25.0	67%	49%	0.60 (0.57–0.63)	< 0.001	27.0	64%	52%
Waist to Hip Ratio	0.64 (0.61–0.68)	< 0.001	0.92	69%	53%	0.67 (0.64–0.71)	< 0.001	0.96	61%	66%
Waist to Height Ratio	0.63 (0.59–0.66)	< 0.001	0.52	70%	52%	0.65 (0.63–0.69)	< 0.001	0.60	72%	55%

*CI* Confidence interval, taking into account the Bonferroni correction (dividing the significance level of 0.05 by the number of comparisons being made), the significance level for a given comparison would be 0.01

^*^Null hypothesis: true area = 0.5

Based on Table 3, anthropometric measures were more accurate in predicting HTN in people aged < 50 than those aged ≥ 50.

**Table 3:** Area under the curve and optimal cut-off points demonstrating the ability of anthropometric indices to predict hypertension according to age

Measures	Age < 50					Age ≥ 50				
	**AUC (99% CI)**	*P* value^*^	**Cut-off-value**	**Sensitivity**	**Specificity**	**AUC (99% CI)**	*P* value^*^	**Cut-off-value**	**Sensitivity**	**Specificity**
Waist Circumference (cm)	0.64 (0.60–0.67)	< 0.001	92.0	69%	54%	0.61 (0.57–0.64)	< 0.001	90.0	75%	43%
Hip Circumference (cm)	0.60 (0.55–0.63)	< 0.001	99.0	67%	47%	0.56 (0.53–0.60)	< 0.001	95.0	68%	43%
Wrist Circumference (cm)	0.60 (0.57–0.64)	< 0.001	17.0	48%	67%	0.56 (0.52–0.59)	< 0.001	16.0	53%	56%
Body Mass Index (kg/m^2^)	0.64 (0.61–0.68)	< 0.001	25.0	79%	43%	0.59 (0.56–0.63)	< 0.001	26.0	63%	52%
Waist to Hip Ratio	0.62 (0.59–0.66)	< 0.001	0.90	75%	43%	0.60 (0.57–0.64)	< 0.001	0.96	58%	58%
Waist to Height Ratio	0.62 (0.59–0.66)	< 0.001	0.60	47%	73%	0.60 (0.56–0.64)	< 0.001	0.60	50%	65%

*CI* Confidence interval, taking into account the Bonferroni correction (dividing the significance level of 0.05 by the number of comparisons being made), the significance level for a given comparison would be 0.01.*Null hypothesis: true area = 0.5

The optimal cut-off points for anthropometric indices were determined according to Youden's J statistic. The optimal cut-off points were developed for WC (90 cm for males and 96 cm for females), HC (95 cm for males and 108 cm for females), WrC (17 cm for males and 15 cm for females), BMI (25 kg/m2 for males and 27 kg/m2 for females), WHR (0.92 for males and 0.96 for females), and WHtR (0.52 for males and 0.60 for female) (Table 2).

The optimal cut-off values for screening hypertension were also determined based on age. The optimal cut-off points were developed for WC (92 cm for age < 50 and 90 cm for age ≥ 50), HC (99 cm for age < 50 and 95 cm for age ≥ 50), WrC (17 cm for age < 50 and 16 cm for age ≥ 50), BMI (25 kg/m2 for age < 50 and 26 kg/m2 for age ≥ 50), WHR (0.90 for age < 50 and 0.96 for age ≥ 50), and WHtR (0.60 for age < 50 and 0.60 for age ≥ 50) (Table 3).

After adjusting for the age, physical activity, socio-economic status, smoking, and hookah effects, the anthropometric indices were dichotomized using the defined cut-off points and then evaluated for their relationships with HTN (Table 4). The odds of HTN were significantly increased for all dichotomized measures. Among males, those who had increased WHR, WHtR, BMI, WC, HC, and WrC were 1.91, 2.24, 2.04, 2.16, 1.87, and 1.55 times respectively, more likely to be hypertensive than those with appropriate indices.

**Table 4:** Relationships between anthropometric indices dichotomized by the established cut-off values and diagnosed hypertension based on multivariable logistic regression

Measures^a^	Male			Female		
	**Odds ratio**	**95%CI**	***P*-value**	**Odds ratio**	**95%CI**	***P*-value**
Waist Circumference (cm)	2.16	1.72–2.71	< 0.001	2.25	1.85–2.73	< 0.001
Hip Circumference (cm)	1.87	1.46–2.37	< 0.001	1.66	1.33–2.07	< 0.001
Wrist Circumference (cm)	1.55	1.24–1.93	< 0.001	1.84	1.47–2.30	< 0.001
Body Mass Index (kg/m^2^)	2.04	1.62–2.58	< 0.001	2.14	1.76–2.61	< 0.001
Waist to Hip Ratio	1.91	1.52–2.40	< 0.001	2.10	1.73–2.56	< 0.001
Waist to Height Ratio	2.24	1.77–2.83	< 0.001	2.61	2.12–3.20	< 0.001

^a^For males, references were Waist Circumference ≤ 90 cm, Hip Circumference ≤ 95 cm, Wrist Circumference ≤ 17 cm, BMI ≤ 25 kg/m2, WHR ≤ 0.92 cm, and WHtR ≤ 0.52 cm. Also, for females, references were Waist Circumference ≤ 96 cm, Hip Circumference ≤ 108 cm, Wrist Circumference ≤ 15 cm, BMI ≤ 27 kg/m2, WHR ≤ 0.96 cm, and WHtR ≤ 0.60 cm. Adjusted for age subgroups, physical activity, socio-economic status, smoking, and hookah consumption

Whereas among females, those who had increased WHR, WHtR, BMI, WC, HC, and WrC were 2.10, 2.61, 2.14, 2.25, 1.66, and 1.84 times respectively, more likely to be hypertensive than those with appropriate indices.

## Discussion

This population-based cross-sectional study aimed to determine optimal cut-off values of several anthropometric indices for the prediction of HTN among southern Iranian adults. Based on ROC analysis, our study demonstrated that WHR and WHtR, as abdominal obesity indices, provided better screening ability for hypertension compared to other measures among both sexes.

Consistently, Feldstein et al. demonstrated that WHR better predicts HTN compared to WC and BMI [35]. Also, Esmaillzadeh et al. concluded that WHR was a better screening measure for cardiovascular risk factors, especially HTN, than other anthropometric indices in adult men [20]. Evidence suggests that WHR is positively correlated with arterial stiffness [36], which plays a significant role in the pathogenesis of HTN and can predict the likelihood of cardiovascular events in hypertensive patients [37]. Moreover, excess abdominal fat deposition causes high-density lipoprotein (HDL) levels to decrease, and low-density lipoprotein (LDL) and triglyceride levels to increase, and a high LDL level is a well-established risk factor for HTN [38]. Additionally, the superiority of WHR may also be due to the fact that WHR incorporates a hip circumference measurement, which is inversely associated with HTN [39].

Furthermore, several studies have also shown that WHtR provides a more reliable prediction of HTN among different populations [29, 40, 41]. A potential explanation for the higher prediction power of WHtR could be that height may have an impact on how fat is distributed, making short individuals more likely to accumulate central fat and have greater SBP levels [42].

Inconsistent with our findings, some cross-sectional studies have indicated that other obesity indices better predict hypertension than WHR and WHtR. For instance, In some studies, BMI and WC have been suggested to better predict HTN [35, 43, 44]. BMI cannot distinguish between fat mass and muscle mass, leading to underestimating health risks; therefore, using only height and weight to screen for health risks is becoming obsolete [45]. On the other hand, although health risks are generally associated with central fat distribution [46], WC is collinear with BMI and weight, and has a limited ability to predict morbidity and mortality [47].

In the current study, the optimal cut-off values established for WHR to detect HTN were 0.92 in males and 0.96 in females, which are higher compared to the WHO-recommended sex-specific WHR cut-offs (0.90 in males and 0.85 in females) [48], and those estimated for an Ethiopian population [22]. Also, cut-off values of WHtR in our study were higher than in previous cross-sectional studies [41]. Additionally, cut-off points for WC to screen for HTN were determined to be 90.0 cm for males and 96.0 cm for females in the current study, while previously established cut-offs were 76–102 cm for males and 75–88 cm for females [24, 48, 49]. Furthermore, we found that BMI cut-offs were 25 kg/m^2^ for males and 27 kg/m^2^ for females. At the same time, cut-offs from other studies ranged from 22.2 to 24.6 for males and 24.3 to 27.7 for females [22, 50, 51]. The discrepancies between studies can be explained by ethnic and racial differences, as well as variations in body composition and fat distribution among different age and sex groups. Different research designs, measurement protocols, or criteria to define cardiometabolic outcomes may also make a difference.

Another noteworthy finding from this study is that anthropometric indices were generally more accurate in predicting HTN in individuals under 50 than those equal to or over 50, as well as females than males. One potential explanation can be the stronger correlation of these indices with HTN in females reflected in the generally higher odds of HTN than males in the logistic regression analysis, even after adjustment for age, physical activity, socio-economic status, smoking, and hookah consumption. Moreover, our previous research on the same population has shown that all metabolically unhealthy states were significantly higher in females than in males [52], and blood pressure is a component of metabolic health. The higher accuracy of indices in females, at least in part, can also account for the higher accuracy in those aged < 50 years since almost two-thirds of females were under 50.

Considering the results of the present study differed based on age and sex, the authors believe that there is a need to develop age and sex-specific cut-off values that are appropriate for different populations, as there is no consensus regarding these values in the literature when it comes to screening hypertension in adults. Furthermore, this study has significance for developing countries such as Iran because of the lifestyle changes caused by urbanization that may impact nutritional status and demographics. As a result of this condition, NCDs prevalence, like HTN and diabetes mellitus, has unexpectedly risen, which in turn leaves the country facing a heavy burden of non-communicable diseases [53]. Therefore, initiatives aiming at preventing NCDs like HTN can be developed using the findings from our study. The use of straightforward, non-invasive anthropometric indices in structured population-based or primary care facility-based HTN screening can easily achieve this.

There were several limitations to this study. Data for this study were taken from a cohort study conducted among southern Iranians aged between 35 and 70, which was a particular study group. It is uncertain if the same results hold true for other age groups or ethnicities. Additionally, due to the study's cross-sectional nature, we were unable to draw causal conclusions about the association between obesity indices and HTN. Another limitation may have been measurement error. Even though we attempted to reduce measurement errors, there is still a possibility of errors occurring. Despite these drawbacks, the results of this study are significant in terms of their implications for the prevention of HTN and the reduction in its occurrence among both males and females in the age group and population investigated. Thus, although the current study adds to the literature, future research that enables establishing a causal link between adiposity indices and HTN in other populations by multicenter longitudinal design is advised.

## Conclusions

In a cohort of Iranian males and females aged 35–70, the measurement of WHR and WHtR, as anthropometric indices of obesity, were significantly indicative of the occurrence of HTN. The results of this study showed the significance of anthropometric obesity indices as predictors of HTN, despite the fact that the study was restricted to the middle-aged Iranian population and had some other limitations. Additionally, we propose using WHR (cut-off point of 0.92 for males and 0.96 for females) and WHtR (cut-off point of 0.52 for males and 0.60 for females) as the measures of preference to predict HTN in Iranians. Finally, we suggest that WHR and WHtR be regularly monitored in the healthcare system and utilized as indicators for evaluating the risk of HTN.

## Data Availability

The datasets used and/or analyzed during the current study are available from the corresponding author on reasonable request.

## Abbreviations

ADA: American Diabetes Association
AUROC: Area Under the Receiver Operating Characteristic
T2DM: Type 2 diabetes mellitus
FPG: Fasting plasma glucose
BMI: Body mass index
WC: Waist circumference
HP: Hip Circumference
WrC: Wrist Circumference
WHR: Waist to Hip Ratio
WHtR: Waist to Height Ratio
SBP: Systolic Blood Pressure
DBP: Diastolic Blood Pressure
BP: Blood pressure
HTN: Hypertension
CI: Confidence interval
WHO: World Health Organization
TG: Triglyceride
TC: Total cholesterol
HDL-C: High-density lipoprotein cholesterol
LDL-C: Low-density lipoprotein cholesterol
BKNCD: Bandare-Kong Non-Communicable Disease
PERSIAN: Prospective Epidemiological Research Studies in IrAN
